# Observation of biological and emulsion samples by newly developed three-dimensional impedance scanning electron microscopy

**DOI:** 10.1016/j.csbj.2024.11.023

**Published:** 2024-11-12

**Authors:** Toshihiko Ogura, Tomoko Okada

**Affiliations:** Health and Medical Research Institute, National Institute of Advanced Industrial Science and Technology (AIST), Central 6, Higashi, Tsukuba, Ibaraki 305-8566, Japan

**Keywords:** Scanning electron microscopy, Impedance SEM, Biological sample, Emulsion, Silicon nitride film, Multi-frequency, 3D reconstruction, Simulated Annealing algorithm

## Abstract

Imaging at nanometre-scale resolution is indispensable for many scientific fields such as biology, chemistry, material science and nanotechnology. Scanning electron microscopes (SEM) are widely used as important tools for the nanometre-scale analysis of various samples. However, because of the vacuum inside the SEM, a typical analysis requires fixation of samples, a drying process, and staining with heavy metals. Therefore, there is a need for convenient and minimally invasive methods of observing samples in solution. Recently, we have developed a new type of impedance microscopy, multi-frequency impedance SEM (IP-SEM), which allows nanoscale imaging of various specimens in water with minimal radiation damage. Here, we report a new IP-SEM system equipped with a linear-array terminal, which allows eight tilted images to be observed in a single capture by applying eight frequencies of input signals to each electrode. Furthermore, we developed a three-dimensional (3D) reconstruction method based on the Simulated Annealing (SA) algorithm, which enables us to construct a high-precision 3D model from the 8 tilted images. The method reported here can be easily used for 3D structural analysis of various biological samples, organic materials, and nanoparticles.

## Highlights

• Newly developed a 3D impedance SEM with a linear array terminal.
• Eight tilted images are captured with one electron beam scan.
• Highly accurate 3D reconstruction is enabled by applying the SA algorithm.
• Our system enables 3D reconstruction of cell and emulsion samples.

## Graphical abstract

**Figure:**
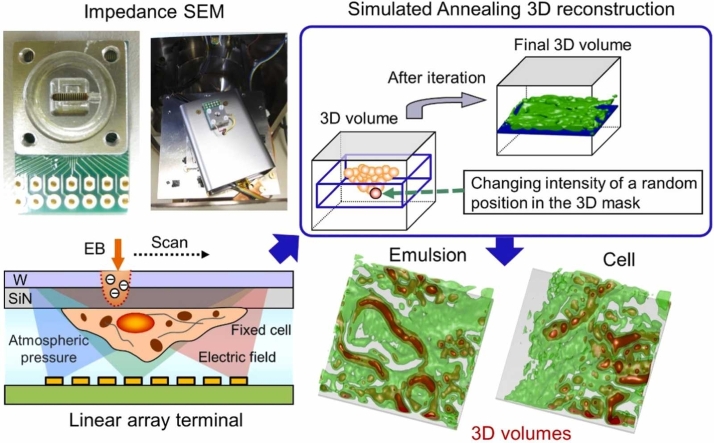

## 1 Introduction

Techniques for observing and analysing organic materials and biological samples at nano-scale resolution are required in various scientific and industrial fields [1], [2], [3], [4]. Transmission electron microscopy (TEM) and scanning electron microscopy (SEM) are commonly used for the high-resolution observation of organic materials and biological samples [5], [6], [7]. However, electron microscopes in general are not suitable for observing samples in solution or under atmospheric pressure conditions because of the vacuum inside the device.

X-ray microscopy has been developed as a method of observing various samples under atmospheric pressure [8], [9], [10]. X-ray microscopy has a higher spatial resolution than optical microscopy because it uses light of a much shorter wavelength. In addition, X-ray tomography microscopes have been developed in which images are acquired at different tilt angles where the sample is rotated to construct a 3D structure [11], [12]. On the other hand, for detailed analysis of the internal structure of organic materials and biological samples, it is important to analyse the chemical composition inside the sample [13]. Infrared spectroscopy and Raman microscopy are used for analysing the composition of samples [13], [14], [15], [16], [17]. These devices can analyse the composition by using the infra-red spectrum and Raman spectrum of each pixel in the observed image. However, since they use light as a probe, their resolution is limited to about 200 nm [18].

Recently, biological samples have been observed using environmental SEM, which observes samples in a low vacuum while changing the sample temperature [19], [20], [21], [22]. By placing biological cells or plants on a temperature-controlled stage and observing them with the SEM in a low vacuum, it is possible to observe them while maintaining moisture [19], [20], [21], [22]. However, it is difficult to observe the internal structure of the cells because the observation is basically by SEM.

We have developed scanning-electron assisted dielectric microscopy (SE-ADM) [23], [24], [25] and impedance scanning-electron microscopy (IP-SEM) [26], [27] based on SEM. SE-ADM enables direct observation of cultured cells and organic materials in solution at nano-level spatial resolution without staining or fixation [17], [28], [29], [30]. In IP-SEM, a sine input signal is applied to the terminal in the lower part of the sample holder, and this signal passing through the sample is detected from the W-layer that coats the top side of a SiN film opposite to the sample side, the upper part of the sample holder [26], [27]. The SiN film is irradiated from the top with an electron beam (EB) during scanning, and the image is generated by measuring the impedance change of the detector. Our observation method allows biological samples and organic matter in solution to be directly observed in their intact state, with low damage caused by the EB [26]. Furthermore, with our impedance microscope, we have succeeded in simultaneously applying sine waves of eight different frequencies to a sample in solution and acquiring an impedance image for each frequency [27].

In this study, the multi-frequency IP-SEM has been improved to enable analysis of the 3D structure of the sample. In this system, the input signal application terminal at the bottom of the sample holder is a linear-array terminal, which enables 3D structural analysis with a single EB scan. Moreover, by using the Simulated Annealing (SA) algorithm [31], [32], a high-precision 3D structure can be obtained from eight tilted images from eight electrodes. In a general computed tomography (CT) method [33], images need to be repeatedly taken while rotating the sample or tilting the stage [34], [35], [36], which results in a long measurement time and accumulated damage to the sample. In contrast, with our method, multiple tilted images can be acquired with a single EB scan, resulting in a short measurement time and correspondingly low damage to the samples.

## 2 Results

### 2.1 Eight-frequency 3D IP-SEM system

**Fig. 1:**
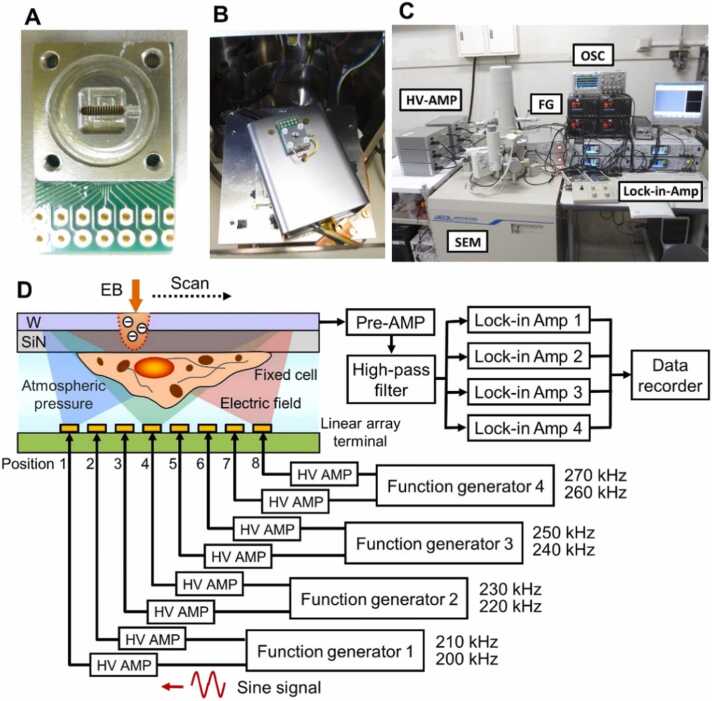
Overview of the 8-frequency 3D IP-SEM system. (A) A photograph of the lower sample holder component with linear array terminals attached. (B) A photograph of the box containing the preamplifier (Pre-AMP) in the SEM chamber and the sample holder mounted on the box. (C) A photograph of the 8-frequency 3D IP-SEM system, with the SEM on the left side and four function generators (FG), four lock-in amplifiers (Lock-in-Amp) and an oscilloscope (OSC) for impedance waveform observation on the desk on the right. Eight high-voltage amplifiers (HV-AMP) are located on the left side of the SEM. (D) Schematic representation of the 8-frequiency 3D IP-SEM system. The 8-frequency signals from the four function generators were input to the eight high-voltage amplifiers. Each signal was introduced to each electrode terminal (Position 1–8) under the sample holder in the SEM chamber via a connector. The signals from the upper W-layer detected were input to the four lock-in amplifiers after passing through the preamplifier and a high-pass filter. The signals separated into eight frequencies by the four lock-in amplifiers were recorded in two data recorders.

Fig. 1 shows an overview of the impedance observation system that enables simultaneous 8-channel tilted image detection. A linear-array electrode terminal including 8 terminals that applies 8 different frequencies is attached to the bottom of the sample, and an electric field is applied to the sample through a long and narrow aluminium aperture in the centre (Fig. 1A). The upper part of the sample holder, to which the SiN film is attached, is sealed with the lower holder by four screws, and the inside is kept at atmospheric pressure. The sample holder containing the sample and the linear-array terminal are placed on the sample stage inside the SEM, which has a built-in preamplifier (Fig. 1B). The linear-array terminal is applied with an eight-frequency sine wave signal from four function generators located on the desk of the SEM via a high-voltage amplifier (Figs. 1C and 1D).

An input sine wave signal of 200–270 kHz is applied from the leftmost electrode to the rightmost electrode of the linear-array terminal (Fig. 1D). The input signal applied to each electrode is transmitted through the sample and propagated to the upper W-layer, where the signal is detected via the electrically coupled upper aluminium sample holder (Fig. 1D). The detected signal contains a mixture of sine wave signals ranging from 200 kHz to 270 kHz frequency. The detected signal is amplified by a preamplifier and passed through a high-pass filter, and the components of the sine wave signals at eight frequencies are extracted by four lock-in-amps (Fig. 1D). Furthermore, an impedance image is constructed from the detected signal at each frequency and the scanning signal of the EB. Here, the impedance image at each frequency provides a tilted image that depends on the angle between the applied electrode and the irradiated position of the EB. For example, the impedance image of a 200 kHz sine wave signal produces a tilted image that depends on the angle between the applied leftmost electrode and the EB irradiation position. The important point here is that the same number of tilted images as the applied input signals can be obtained simultaneously in a single EB scan. Further, since there is no need to tilt the sample stage, the observation time is shorter and sample damage is low compared to conventional CT.

### 2.2 Observation of polystyrene spheres by 3D IP-SEM

**Fig. 2:**
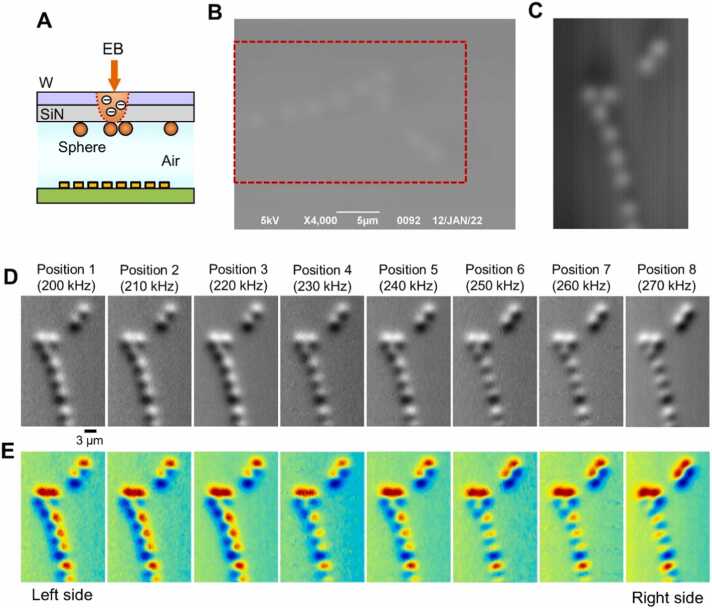
Image comparison between SEM and IP-SEM. (A) Schematic diagram of the PS sphere observation method. The spheres are attached to the underside of the W-coated SiN film, and the EB is incident on the upper tungsten layer. (B) A secondary electron image of 3 µm PS spheres attached to the SiN film by SEM, scanned at a magnification 4000 × and 5 kV EB. (C) A secondary electron image of PS spheres was rotated 90° counterclockwise, the red rectangle region of (B) was cut out and the contrast was enhanced. (D) Eight IP-SEM images of PS spheres when sine wave signal was applied from 200 kHz to 270 kHz in 10 kHz steps from the left input terminal to the right terminal. (E) Pseudo-colour images of (D). Scale bars, 5 µm in (B), 3 µm in (D).

First, a 3 µm polystyrene (PS) sphere sample was observed with this system (Fig. 2). The spheres were attached to a 50 nm-thick SiN film and the SEM secondary electron image and impedance image were obtained simultaneously (Fig. 2A). Fig. 2B shows a secondary electron image of the PS sphere, in which a row of spheres is faintly visible.

Under these observation conditions, the sample is partially irradiated by the EB under the tungsten-coated SiN film, and secondary electrons are emitted when these scattered electrons pass through the tungsten layer. The SE detector detects and creates images from these secondary electrons [37].

The scanning direction of the EB and the arrangement of the linear-array terminal were placed at a right angle to each other, and the scattered electron image was rotated 90° counterclockwise to correspond with the impedance microscopy image to cut out the region of the spheres and enhance the contrast (Fig. 2C).

Fig. 2D shows impedance amplitude images of the PS spheres at eight frequencies. A 200 kHz input signal was applied to the leftmost electrode, and the input signals were added to each of the electrodes to the right in 10 kHz steps. The image at each frequency was tilted according to the angle between the applied array electrode and the irradiation position of the scanning EB. Therefore, the image of the leftmost electrode was bright on the right side of the spheres and dark on the left side. On the other hand, the IP-SEM image of the right electrodes show a gradual change in luminosity (Figs. 2D and 2E).

We calculated the spatial resolution of our IP-SEM system and SEM using the edge images (Supplementary Fig. 1A and 1B). To measure spatial resolution, the averaged output across the SiN edge is shown in Supplementary Fig. 1C–1F. The spatial resolutions of SEM and IP-SEM are 207 nm and approximately 250 nm, respectively, defined as the distance over which the normalized intensity decreases from 0.75 to 0.25 in the output. The resolution of the IP-SEM is slightly lower than that of the SEM secondary electron image. This is probably because the IP-SEM electrode is in an inclined position and has a broad shape.

### 2.3 Observation of sunscreen lotion sample

**Fig. 3:**
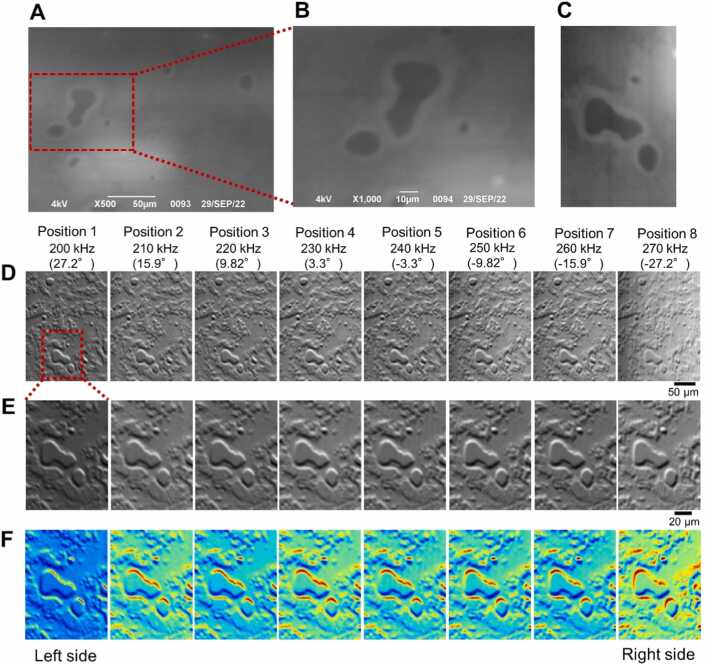
Image comparison of sunscreen lotion sample between SEM and IP-SEM. (A) A low-magnification SE image of the sunscreen lotion sample attached to the SiN film by SEM, scanned at a magnification 500 × and 4 kV EB. (B) A 1000 × magnification SEM image of the sunscreen lotion from the left side of (A). (C) A secondary electron image of sunscreen sample was rotated 90° counterclockwise, the red rectangle region of (A) was cut out and the contrast was enhanced. (D) Eight low-magnification (500 ×) IP-SEM images of sunscreen lotion when sine wave input signal was applied from 200 kHz to 270 kHz in 10 kHz steps from the left terminal to the right terminal. (E) 1000 × magnification eight IP-SEM images of sunscreen lotion in red rectangle of (D). (F) Pseudo-colour images of (D). Scale bars, 50 µm in (A) and (D), 10 µm in (B), 20 µm in (E).

Next, we observed sunscreen lotion using a 3D IP-SEM system (Fig. 3). Sunscreen lotion was applied to the bottom surface of the SiN film, sealed in an atmospheric sample holder, and observed using a secondary electron detector and IP-SEM. Secondary electron images revealed dark circles and a gourd-shaped structure surrounded by white areas (Fig. 3A–3C). However, it was difficult to obtain the characteristics of the 3D structure from this image alone. In the impedance amplitude image of eight terminals acquired simultaneously, a high impedance region can be seen around the central circular or gourd-shaped structure (Fig. 3D). In this outer periphery, the impedance value is high at the upper edge and low at the lower edge, and this value changes depending on the imaging tilt angle of its electrode (Figs. 3E and 3F).

**Fig. 4:**
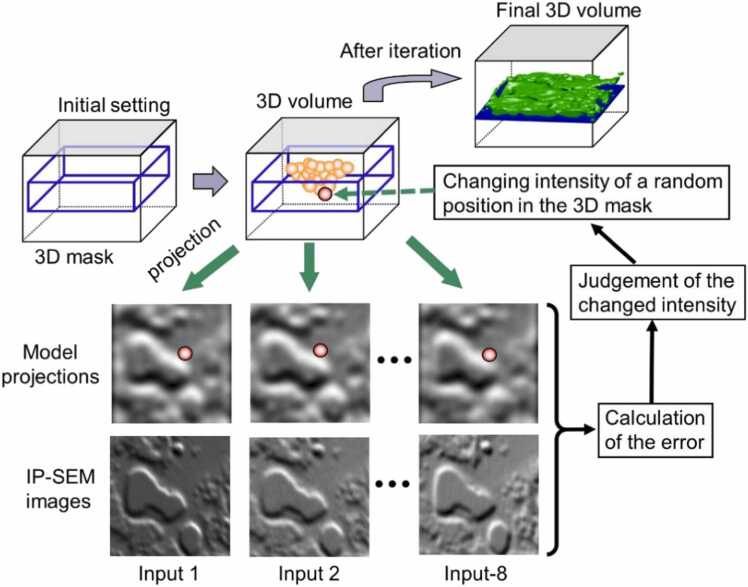
Schematic diagram of the 3D reconstruction method using the SA algorithm with IP-SEM images. In the initial setting, all values in the areas that construct the 3D structure of the sample were set to 0. Next, we selected a random position in the sample area, changed its voxel values in a random shift, calculated the new error from the new 3D volume and fed this new error to the SA algorithm to determine whether to accept the modification. The error value used to determine whether to accept the modification was the residual sum of the squares of the projections from the new 3D volume and its corresponding IP-SEM images. This process was iterated for a predetermined number of times (100,000 times for the work reported here). The temperature and kernel of changing voxels were thereby gradually reduced. These steps were iterated for a predetermined number of cycles (here 5000 cycles).

We further constructed the 3D structure from eight tilted impedance images. Generally, in 3D reconstruction from tilted images, the 3D structure is obtained using weighted back projection or the simultaneous reconstruction technique [38], [39]. However, because only eight images could be observed in this system and the observation angle was also narrow at ± 27 degrees, it is difficult to accurately calculate the 3D structure using conventional methods. Therefore, in this study, we developed a new 3D reconstruction algorithm for IP-SEM using the SA algorithm (Fig. 4).

### 2.4 Simulated Annealing 3D reconstruction algorithm

We have previously developed an X-ray microscope system that uses the X-ray linear array device including 7 X-ray detectors to obtain seven tilted images to determine the 3D structure of bacteria [32]. To reconstruct highly accurate 3D structures from a small number of tilted images, we have developed a 3D reconstruction method that applies the SA algorithm [32]. Here, we applied this 3D reconstruction method using the SA algorithm to the tilted images of the IP-SEM to produce the 3D structure (Fig. 4). Firstly, the algorithm sets an empty space (all zero values) as an initial value, selects a random position in this 3D space and slightly changes its intensity. From this 3D model with a changed intensity value, a projection image is calculated for each of the eight impedance tilted images at the corresponding angles. The total error is calculated from the sum of the squares of the differences between the eight projection images and the corresponding IP-SEM images (see 3D reconstruction algorithm under Materials and Method). If the current error value is lower than the previous error value, this intensity change is determined. If the current error value is higher, then the decision to confirm or reject is made probabilistically according to the Boltzmann distribution with the current temperature [31], [32]. These cycles are repeated a predetermined number of times to gradually reduce the virtual temperature and the range of the changing 3D structure space.

**Fig. 5:**
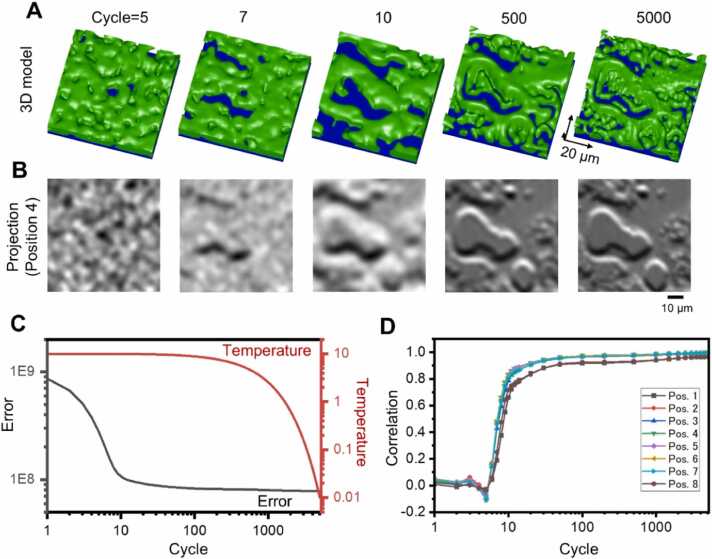
3D reconstruction from tilted IP-SEM images of a sunscreen lotion sample using the SA algorithm. (A) Evolution of the 3D structure with increasing annealing cycles. Initially, the 3D volume is blank. Starting from the blank volume, the 3D volume rapidly emerged at just 10 cycles. Beyond 500 cycles, the 3D volume was gradually optimized, and the fine structure appeared. The length of the arrow in each 3D direction is 20 µm. (B) Changes in the projected image of the central terminal position (Position 4 in Fig. 3E) while the number of annealing cycles increased. (C) Change in temperature and error with increasing annealing cycles. (D) Change in cross-correlation values between each re-projection image (terminal Position 1–8) and the IP-SEM tilted image with increasing annealing cycles. Scale bars, 10 µm in (B).

**Fig. 6:**
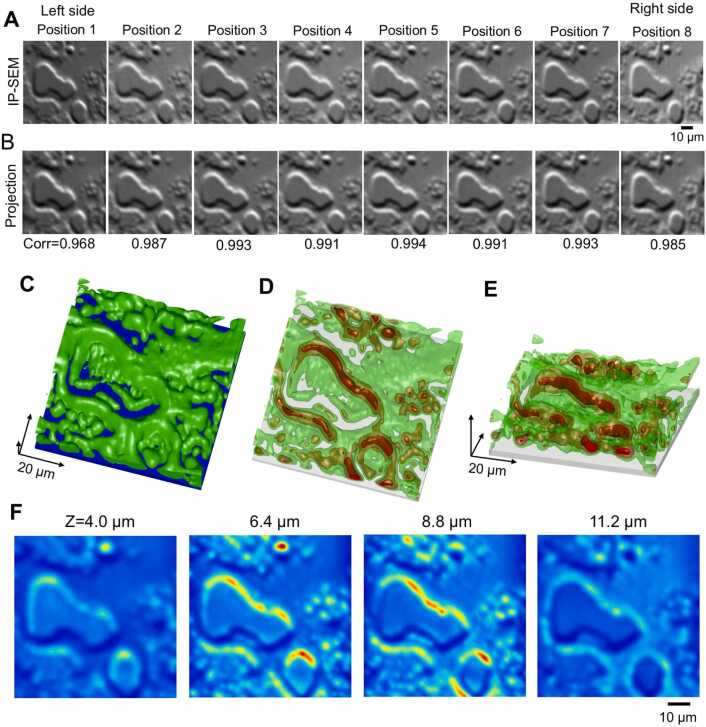
Analysis of the final structure of the sunscreen lotion sample using the SA algorithm. (A) The IP-SEM images of Fig. 3E used for 3D reconstruction. Eight images with input frequencies from 200 to 270 kHz. (B) Projection images of the final 3D structure using the SA algorithm at the corresponding tilt angles. (C) Image of the final 3D structure of the sunscreen lotion sample. The green areas indicate the sunscreen lotion structure, and the blue areas, the SiN thin film. In this 3D map, the sample was rotated upside down so that the SiN film was at the bottom and the sample was at the top to make it easier to see. The length of the arrow in each 3D direction is 20 µm. (D), (E) Transmission images of high-density areas within 3D structures (red areas). Viewing angle was 60 degrees for (D) and 30 degrees for (E). (F) Impedance map sliced along the XY axis. The boundary with the SiN film is set to Z = 0 µm, and four map images at 4.0 – 11.2 µm above (4.0 to 11.2 µm below in the original 3D position) the SiN film are shown here. Scale bars, 10 µm in (A) and (F).

Figs. 5A and 5B show changes in the 3D structure and projection images when 3D reconstruction was performed with this algorithm using the IP-SEM images of the sunscreen lotion in Fig. 3E. When the calculation cycle was 5, only noise components were seen in both the 3D structure and the projection image (Figs. 5A and 5B, Cycle=5). At cycle 7, a structure with dark horizontal stripes was formed in the centre and at the top, and at cycle 10, a blurred overall structure could be seen (Figs. 5A and 5B, Cycle=7 and 10). At cycle 500, the structure was very similar to the original structure, after which more detailed refinement was carried out (Figs. 5A and 5B, Cycle=500). At the final cycle of 5000, the projection image was almost consistent with the original IP-SEM image (Figs. 5A and 5B, Supplementary Fig. 2). Fig. 5C shows the virtual temperature and error for this calculation. The error decreased rapidly between cycles 1 and 10 and continued to decrease gradually thereafter, almost saturating at cycles 1000 and above. In the cross-correlation values between the eight IP-SEM images and each model projection image at each cycle, the cross-correlation values remained around 0 until cycle 7, but the correlation values increased rapidly from cycle 7 to 12, and the increase became very gradual after cycle 100 (Fig. 5D). At the end of cycle 5000, correlation values of all position images were above 0.95 (Fig. 5D), confirming that the final projection images were extremely similar to the original IP-SEM images (Figs. 6A and 6B).

The 3D structure at the end of the calculation is shown in Figs. 6C–6E. A low impedance groove was present below the central gourd-shaped structure and a high impedance contour structure was found around it (Fig. 6C). Moreover, in the centre of the contour structure, a region with high impedance values was observed along the contour (Figs. 6D and 6E, red areas). In the map image of the XY-axis slice in the depth direction from the SiN film, a noticeable high impedance area was seen on the outer periphery of the gourd-shaped structure in the map at a depth of 6.4 and 8.8 µm (Fig. 6F). This suggests that oil components were adhering to the periphery.

### 2.5 Observation and 3D reconstruction of MNT-1 cells

**Fig. 7:**
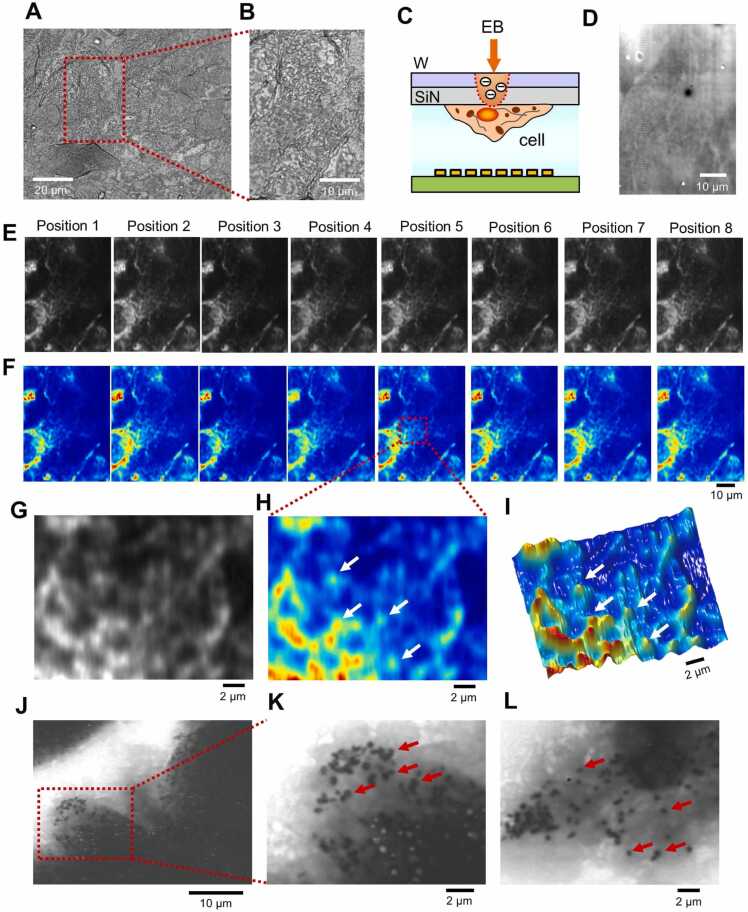
Image comparison of cell sample between optical microscopy, SEM and 3D IP-SEM. (A) Optical microscope image at 1000 × magnification of MNT-1 cells dried after chemical fixation. (B) Enlarged image of the red rectangle area in (A). (C) Schematic diagram of cell sample observation. The cells are attached to the underside of the W-coated SiN film, and the EB is incident on the upper tungsten layer. (D) SEM image of secondary electrons at the same position as in (B) at a magnification 2000 × and 5 kV EB. (E) IP-SEM images of each position of the 8 electrodes. (F) Pseudo-colour image of (E). Areas with high impedance values can be seen in the cell nucleus and its surrounding areas. (G, H) Enlarged and pseudo-colour images of the red rectangle in Position 5. The white arrow indicates a particle with high impedance value. (I) Pseudo 3D image in (H). (J-L) Images of MNT-1 cells dried after chemical fixation by SE-ADM. The red arrows indicate melanosomes. Scale bars, 20 µm in (A), 10 µm in (B), (D), (F) and (J), 20 µm in (G-I).

We next observed cultured cells by IP-SEM and performed 3D reconstruction. Melanin pigment-producing melanoma cells (MNT-1) were cultured on SiN membranes, chemically fixed with paraformaldehyde (PFA), dried and observed by SEM (Fig. 7).

In the optical microscope images, the outlines of the cells and protruding structures can be observed (Figs. 7A and 7B). The same area as in Fig. 7B was observed simultaneously by IP-SEM and secondary electrons (Figs. 7C–7F). In this observation, the cell structure of secondary electrons is not clear because the cells are attached to the underside of the SiN film (Fig. 7C) and there is not enough secondary electron generation in the tungsten layer due to reflected electrons from the cells (Fig. 7D). Furthermore, in this observation, the number of secondary electrons associated with the reflected electrons is lower because the current of the EB is low, 200 pA. On the other hand, as the current value of the EB is decreased, the probe diameter of the EB from the thermal electron gun becomes smaller, and the cell structure becomes clearer in the IP-SEM images (Figs. 7E and 7F). When the area around the cell nucleus in the IP-SEM image of the central electrode 5 was enlarged, many particles with high impedance values were seen (Figs. 7G–7I). When fixed and dried MNT-1 cells were observed with SE-ADM (Figs. 7J–7L), many black particles of melanosomes were observed, just as is seen in live cells [17], [30]. Therefore, the particles observed in IP-SEM are also considered to be melanosomes.

Our method makes it possible to simultaneously obtain eight tilted IP-SEM images using eight electrodes. This results in less EB damage than taking eight images while tilting the sample. To confirm this, the same location was scanned eight times, and the change in the image and the image impedance value were calculated (Supplementary Fig. 3). In the second scan, the contrast is slightly reduced, but almost the same structure is maintained. However, from the fourth scan onwards, the contrast of the high impedance area around the cell nucleus is significantly reduced (Supplementary Fig. 3A). The average impedance value of the entire image decreases linearly up to the sixth scan, and then remains almost constant at 1.7 MΩ (Supplementary Fig. 3B). This suggests that the cell is gradually damaged with each scan, and from the sixth scan onwards, only the strong structure remains, and no further damage accumulates. Even in optical microscope images of the same location, the structure of the cell changes significantly after eight scans (Supplementary Fig. 3C). Therefore, the method of acquiring eight tilted images in a single scan allows for observations that cause less damage to the sample.

**Fig. 8:**
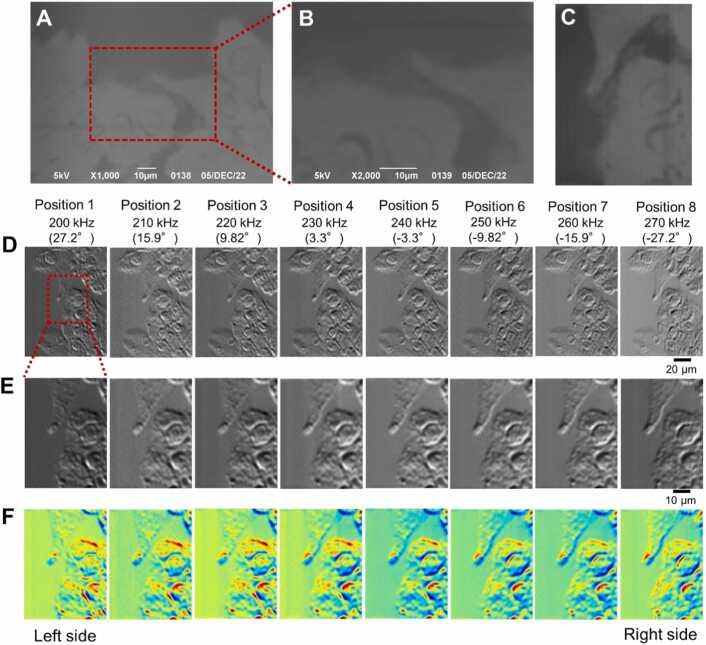
Image comparison of cell sample between SEM and 3D IP-SEM. (A) A low-magnification SEM image of dried MNT-1 cells attached to the SiN film, observed by SEM, and scanned at a magnification 1000 × and 5 kV EB. (B) A 2000 × magnification SEM image of the MNT-1 cells in the red rectangle of (A). (C) A secondary electron image of MNT-1 was rotated 90° counterclockwise, the red rectangle region of (A) was cut out and the contrast was enhanced. (D) Eight IP-SEM images of MNT-1 cells when a sine wave input signal was applied from 200 kHz to 270 kHz in 10 kHz steps from the left terminal to the right terminal (low magnification, 500 ×). (E) 1000 × magnification 8 IP-SEM images of the cells in red rectangle of (D). (F) Pseudo-colour images of (E). Scale bars, 10 µm in (A), (B) and (E), 20 µm in (D).

**Fig. 9:**
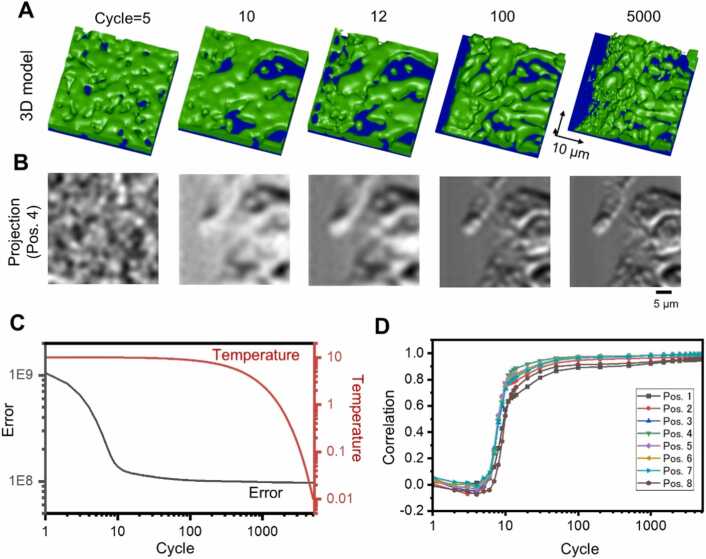
3D reconstruction from tilted IP-SEM images of the MNT-1 cells by the SA reconstruction algorithm. (A) Evolution of the 3D structure with increasing annealing cycles. Initially, the 3D volume was blank. Starting from the blank volume, the 3D volume rapidly emerged in just 10 cycles. Beyond 100 cycles, the 3D volume was gradually optimized, and the fine structure appeared. The length of the arrow in each 3D direction is 10 µm. (B) Changes in the projection image of the central terminal position (Position 4 in Fig. 8E) while the number of annealing cycles increased. (C) Plots of the change in temperature and error with increasing annealing cycles. (D) Change in cross-correlation values between each projection image (terminal Position 1–8 in Fig. 8E) and the IP-SEM tilted image with increasing annealing cycles. After the 10 cycles, the cross-correlation value increased rapidly. At the end of the 5000 cycles of calculation, the cross-correlation value was 0.95 or higher at all terminal positions. Scale bars, 5 µm in (B).

In the SEM observation, when the current of the EB was increased to 600 pA, the outline of the cell is more clearly defined in the secondary electron image (Fig. 8A–8C). However, the internal structure of the cell has low contrast and is not clear (Fig. 8A–8C). Therefore, the detailed structure inside the cell could not be observed. On the other hand, IP-SEM allowed clear observation of the nuclei and granular structures inside the cells (Fig. 8D–8F). In addition, the IP-SEM images at each frequency yielded a tilted image that depends on the angle between the incident input terminal and the scanning EB. 3D reconstruction was performed by the SA reconstruction method using these eight tilted images of cells (Fig. 9).

In the 3D reconstruction of the MNT-1 cell, the cell appeared noisy at cycle 5, but a rough structure corresponding to the cell outline was formed after cycle 10, and a detailed structure inside the cell was observed after cycle 100 (Figs. 9A and 9B). At the final cycle 5000, the fine structure inside the cell was also reproduced, and the projection image in the central part gradually resembled the original impedance image (Figs. 9A and 9B, Supplementary Fig. 4). The changes in the virtual temperature and error with the number of cycles are shown in Fig. 9C, demonstrating that the overall cross-correlation value improved rapidly after 10 cycles (Fig. 9D).

**Fig. 10:**
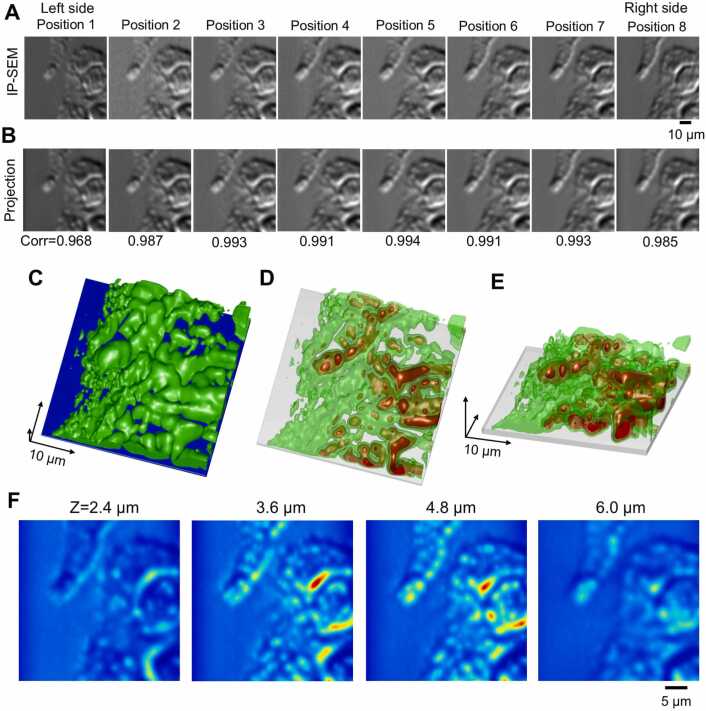
Analysis of the final structure of the MNT-1 cells using the SA algorithm. (A) The IP-SEM images used for 3D reconstruction of Fig. 8. Eight IP-SEM images with input frequencies from 200 to 270 kHz. (B) Projection images of the corresponding tilt angles from the final 3D structure of MNT-1 cells using the SA algorithm. (C) Image of the final 3D structure of the MNT-1 cells. The green areas indicate the cell structure, and the blue areas indicate the SiN film. In this 3D map, the sample was flipped upside down so that the SiN film was at the bottom and the sample was at the top. The length of the arrow in each 3D direction is 10 µm. (D), (E) Transmission 3D images of high-density areas within 3D structures (red areas). Viewing angle was 60 degrees for (D) and 30 degrees for (E). (F) Impedance map sliced along the XY axis. The boundary with the SiN film is set to Z = 0 µm, and four map images at 2.4 – 6.0 µm below the SiN film are shown here. Scale bars, 10 µm in (A), 5 µm in (F).

The original IP-SEM images and the projection images by the final 3D structure were very similar (Figs. 10A and 10B). In addition, the cross-correlation values between the tilted image of each IP-SEM and the projection image of the 3D reconstruction were very high, ranging from 0.96 to 0.99. The 3D structure of the cell at the end of the calculation and the transmission 3D structure confirmed the bulging of the projection part of the cell and the presence of many granules with high impedance values inside cells (Fig. 10C–10E). Furthermore, numerous granules were also present in the cytoplasm in the bottom right-hand corner. This cell granule structure can be clearly observed even on an XY-axis slice map at depths of 3.6 and 4.8 µm (Fig. 10F). These granules are predicted to be melanosomes [17], [30].

## 3 Discussion

We have developed an IP-SEM that enables multiple tilted images of a sample to be observed in a single scan of an EB. In this system, the linear-array terminal to which the input signal of the impedance SEM was applied was used to acquire eight tilted images that depended on the angle between the electrodes and the irradiated EB position by applying sine wave signals of different frequencies to each electrode.

Here, the spacing between the electrodes is narrow at 0.3 mm, so it is possible that there is a crosstalk effect. When the crosstalk signal to the adjacent electrodes was measured, it was less than 10 mV. This is less than 1/10,000 of the 200 Vpp (peak to peak voltage) applied to the electrode, so the effects of crosstalk appear to be small.

Signal amplification may cause stripe noise in the secondary electron image of an SEM when observed with a low-acceleration EB. For example, in Fig. 7D, faint stripe noise can be seen in the SEM image taken with an EB with an acceleration voltage of 5 kV. This noise is more noticeable with an EB with a lower acceleration voltage. This may be caused by the direct effect of the input signal on the EB and its effect on the SE signal. Further, the influence of artifacts due to the dwell time of the scanning EB per pixel should also be considered. In this observation, the time for one scan was 80 s, and the image size was 1280 × 960 pixels, so the dwell time of the EB per pixel was 65 μs. Therefore, its residence time is relatively short and the effects on image deterioration are minor.

A comparison with common 3D structure analysis methods such as X-ray CT, FIB-SEM, and cryotomography is shown in Supplementary Table 1. X-ray CT allows observation under atmospheric pressure, but the observation time ranges from 20 min to several hours because hundreds of images are taken while tilting the specimen [40], [41], [42], [43]. In addition, the resolution is on the submicrometric level. Furthermore, because the specimen is rotated, correction of sample drift and position is necessary. In FIB-SEM, the sample is embedded in resin, observed while being scanned with an ion beam, and 3D reconstruction is performed from these images [44], [45]. The sample is placed in a complete vacuum, and the observation time is approximately one day. On the other hand, cryotomography achieves extremely high resolution because ice-embedded samples or thin-layered samples are observed with a STEM while being tilted [46]. However, sample drift and position correction during tilting are required, and EB damage also occurs. In conventional 3D reconstruction methods, several dozen projection images are needed with tilting of the sample [34], [35], [36], which requires a long time to acquire the images.

Our method can simultaneously acquire multiple tilt images in one scan, shortening the observation time and reducing EB damage, and also preventing sample drift and position shift due to tilt. Therefore, our method is simpler, faster, and less damaging than conventional methods.

Eight tilted images can be acquired in a single EB scan, from which a high-precision 3D structure can be reconstructed. In the reconstructed 3D structures from IP-SEM tilted images of sunscreen lotion and MNT-1 cells, several areas with high internal impedance values were observed, which may indicate aggregates of oil or highly insulating proteins (Fig. 6, Fig. 10). These 3D features of the samples are difficult to analyse from 2D images alone and are an example of the superiority of 3D structural analysis.

To investigate the EB damage caused by repeated scanning with this system, the same area of an MNT-1 cell sample was observed eight times (Supplementary Fig. 3). The results showed that the shape remained almost the same until the second scan, but the impedance of the sample decreased from the fourth scan onwards, and the contrast of the impedance image decreased. Therefore, the effectiveness of a single image taken by our method is high.

If a sample in solution is directly sealed in a sample holder, impedance value drift and noise will take place due to the movement of the sample in solution and due to changes in the thickness of the sealed sample. Therefore, in this study, we used cells that had been dried or sunscreen lotion attached to a SiN membrane as a sample. In the future, we plan to develop a new sample holder that can stably hold a sample in solution and reduce noise components, making it possible to observe live cells, bacteria, etc. We believe that this method will be the first step in providing a new perspective for observing biological samples in the future. Furthermore, we aim to achieve higher accuracy in 3D reconstruction by increasing the number of input signal terminals to 10 or more and using 10 or more tilted images.

Direct observation of nanoparticles and nanostructures is extremely important in the industrial and energy sectors [47], [48], [49], [50], in medical and biological fields [51], [52], [53], [54], and in the analysis of pollution in the environment [55], [56]. Our 3D IP-SEM system is expected to contribute to the direct observation and analysis of various nanoparticles and biological samples without staining and/or labelling.

## 4 Conclusion

We have developed an IP-SEM system with a linear-array terminal. IP-SEM observations were performed by applying a sine wave signal with different frequencies to each of the eight terminals of the linear-array terminal at the bottom of the sample, and eight impedance images with different tilt angles were obtained by frequency separation using four lock-in amplifiers. Furthermore, an SA-applied reconstruction algorithm was used from these eight impedance images to enable high-precision 3D reconstruction. Using this method, it will be possible to analyse the 3D internal impedance values of various materials and biological samples. Our method will be useful in various fields of biology, chemistry, and nanotechnology.

## 5 Materials and methods

### 5.1 Eight-frequency 3D IP-SEM system

The one-dimensional 8-frequency IP-SEM system based on thermionic-SEM (JSM-6390, JEOL, Japan) is shown in Fig. 1. On the underside of the sample holder, electrodes of 0.3 mm width were formed in the linear array terminal, and a sine wave input signal of different frequency was applied to each of these eight central electrodes (Figs. 1A and 1B). The tilt angles of the EB incident positions and the eight terminals are, from left to right, 27.2, 15.9, 9.8, 3.3, −3.3, −9.8, −15.9 and −27.2°.

The input signal was constructed from sine wave signals of eight frequencies generated from four function generators, each of which includes two modules (WF1967, NF Corp., Japan) (Figs. 1C and 1D). Each of the eight-frequency signals was amplified 10 times through a high-voltage amplifier (10 ×, T-HVA03, Turtle Industry Co. Ltd., Japan) and then applied to the metal terminal under the sample holder in the SEM chamber (Fig. 1C). The output signal was detected from the W-layer on the SiN film after passing through the sample (Fig. 1D). This signal was amplified by a preamplifier of the trans-impedance amplifier (200 kΩ gain) located under the sample holder and distributed to four lock-in amplifiers (LI5660, LI5655 and two units of LI5650, NF Corp., Japan) after passing through a high-pass filter (10 kHz cutoff frequency). Each lock-in amplifier has two modules that separate the eight frequencies from the detected signal and output the phase and amplitude of each frequency signal. These output signals and EB scan signals were recorded by two data recorders with 50 kHz sampling frequency (EZ7510, NF Co., Japan).

The SEM images (1280 ×960 pixels) were captured at 200 × to 10,000 × magnification. The scanning time and working distance were 80 s and 7 −8 mm, respectively. The EB acceleration voltage was 4 −7 kV, and the EB aperture was set to be 40 −60 at an operating current of 200 −600 pA.

### 5.2 Metal deposition on the upper SiN film

A 50 nm-thick SiN film supported by a Si frame (area, 4.5 ×4.5 mm with a 0.4 ×0.4 mm window; thickness, 0.381 mm, Silson Ltd., UK) was coated with tungsten using a magnetron sputtering device (Model MSP-30T, Vacuum Device Inc., Japan). Tungsten was sprayed for 10 s under 1.0 Pa Argon with a current of 200 mA, producing a 10 nm-thick coating. The distance between the sputter target and the SiN film was 50 mm [20], [21].

### 5.3 Sample preparations

The PS sphere suspension (Micromer 3 µm, Micromod Co., Germany) was diluted tenfold with pure water. A 5 μL diluted suspension of the PS spheres was placed on a SiN film. Then, the suspension liquid was absorbed by a piece of filter paper and dried at 23 ℃. After drying, spheres attached to the SiN film. The Al sample holder with the spheres was sealed in the sample holder using four screws and an O-ring [23], [24], [25].

In the case of the sunscreen lotion samples (Sun play super cool, ROHTO Pharmaceutical Co. Ltd., Japan), a 5 μL sample was placed on a SiN film. Then, the Al holder with the sample was placed in a centrifuge holder and centrifuged at 5000 rpm for 1 min (MX300, Tommy Co., Japan) to thinly spread the sample on the SiN film.

### 5.4 Cell culture

MNT-1, a human melanoma cell line [57], was obtained from American Type Culture Collection (ATCC) (CRL-3450). MNT-1 cells were cultured in D-MEM (Thermo Fisher Scientific #11995065) containing 10 % AIM-V (Thermo Fisher Scientific #31035025), 10 % MEM NEAA (Thermo Fisher Scientific #10370021), and 20 % fetal bovine serum (Thermo Fisher Scientific) under normal cell culture conditions (37 °C, 5 % CO_2_). Cells (5 × 10^4^, 1.5 mL/dish) were cultured on a 50 nm thick SiN film in a culture dish holder [17], [30]. After 3 −4 days, the cells formed a sub-confluent monolayer on the SiN film in the holder. The cells on SiN film were fixed by 4 % PFA (FUJIFILM Wako Pure Chemical Co.) for 10 min and washed twice with water before being dried at room temperature. Next, the Al holder with a cell monolayer was separated from the plastic culture dish, attached upside down to another SiN film on an acrylic plate and sealed.

### 5.5 High-resolution SE-ADM system

The general-purpose SE-ADM system was attached to a field-emission (FE) SEM (SU5000, Hitachi High-Tech Corp., Japan) [25]. The atmospheric sample holder was mounted on the SEM stage, and the detector terminal was connected to a pre-amplifier in the stage. The electrical signal from the pre-amplifier was fed into the external input of the SEM. The SEM images (1280 × 1020 pixels) were captured at a magnification of 4000–5000 × with a scanning time of 40 s, working distance of 7 mm, an EB acceleration voltage of 3.6 kV, and a current of 1–10 pA. The original SE-ADM images were filtered using a 2D Gaussian smoothing filter (GF) with a kernel size of 11 × 11 pixels and a radius of 1.2σ pixel.

### 5.6 Image processing

The IP-SEM signal data of amplitude and phase stored in the data recorder were transferred to a personal computer (Intel Core i7, 3.8 GHz, Windows 10) and the IP-SEM images were processed by the image processing toolbox in Matlab R2023a (Math Works Inc., USA) [26], [27]. The original IP-SEM images were filtered through a two-dimensional Gaussian filter (GF) with a kernel size of 7 × 7 pixels and a radius of 1.2σ. The background was removed by subtracting the IP-SEM images from the filtered images using a broad GF (kernel size 201 ×201 pixels, radius 100σ). Finally, amplitude and phase images were converted to 8-bit grey scale format.

### 5.7 3D reconstruction algorithm

To generate a 3D model from eight IP-SEM images, we developed a highly accurate reconstruction method using an SA algorithm (Fig. 4) that was implemented by a Matlab script. Previously, we used the SA algorithm for the 3D reconstruction algorithm of the scanning electron X-ray microscope [32] and applied this method to the IP-SEM reconstruction algorithm. Initially, the 3D voxel values, and all projections were set to 0. Starting from the initial value of 0, a position in the 3D voxel was randomly selected, and the voxel values in the kernel area at the selected position were randomly changed (Fig. 4). The kernel area was set to a 3D normal distribution with SD = 15σ, and the randomly changed value in the kernel was SD = 0.2σ, which was gradually reduced with each calculation cycle.

The new 3D volume was reprojected onto the eight directions corresponding to the 8 angles of the IP-SEM terminals, and the error between each reprojection and its corresponding IP-SEM image was calculated by the residual sum of the squares. This calculated error was used by the SA algorithm to determine whether to accept the modified volume [32]. If the new error value was less than the previous error value, the modified volume was accepted unconditionally. If the error had increased, the Boltzmann probability factor of *P*(Δ*E*) was calculated using Eq. (1) and was used to determine whether to accept the modified voxel value.

(1) $$P(\Delta E)=\exp\left(\frac{-\Delta E}{T}\right)$$

In Eq. (1), Δ*E* is the change in the error and *T* is the current virtual temperature. A random number (*R*) uniformly distributed in the interval from 0 to 1 was generated and the volume change was accepted if *R* < *P*(Δ*E*). If *R* > *P*(Δ*E*), the volume was returned to its previous state.

Next, a new position was selected at random, its volume was randomly shifted and the SA algorithm was applied again. This procedure was iterated 100,000 times. When finished, the temperature, the kernel size and its value-shifting SD decreased exponentially. This entire cycle was repeated 5000 times. The temperature, the kernel size and its shifting value were initially set to 10, 15σ and 0.2σ, respectively. After the algorithm had been executed, these values were reduced to 0.1, 2σ and 0.1σ, respectively.

In this calculation, a 3D space with dimensions of 240 pixels in length, width and height is used, and the total number of voxels is 13,824,000. To construct a highly accurate 3D structure, more than 10 iterative calculations are required per voxel. The number of iterative cycles is 5000, with a total of 100,000 × 5000 iterations. Therefore, an average of 36 calculations are performed per voxel, which is considered to be sufficient. The SA algorithm calculation took about 20 h on a Windows 11 pro PC with an Intel Core™ i9–10900X (3.7 GHz) CPU and 128 GB memory. If parallelization were to be performed using a GPU, the speed could probably be increased by more than 10 times [58].

## Funding

This study was supported by Japan Science and Technology Agency CREST Grant Number JPMJCR19H2, Japan Society for the Promotion of Science KAKENHI Grant Numbers 24H00411.

## CRediT authorship contribution statement

**Tomoko Okada:** Writing – review & editing, Writing – original draft, Visualization, Validation, Formal analysis, Data curation, Conceptualization. **Toshihiko Ogura:** Writing – review & editing, Writing – original draft, Visualization, Validation, Supervision, Software, Resources, Project administration, Methodology, Investigation, Funding acquisition, Formal analysis, Data curation, Conceptualization.

## Declaration of Competing Interest

The authors declare that they have no known competing financial interests or personal relationships that could have appeared to influence the work reported in this paper.

## Data Availability

All data generated or analysed during this study are presented in this paper or in the Supplementary Information. All the raw data files or spectra are available from the corresponding author on reasonable request.
