# Supplementary material for: Observation of biological and emulsion samples by newly developed three-dimensional impedance scanning electron microscopy
Source: Comput Struct Biotechnol J. 2024 Nov 12;23:4064–76. doi: 10.1016/j.csbj.2024.11.023 (PMC11613192; doi:10.1016/j.csbj.2024.11.023)

**Supplementary data**

**Observation of biological and emulsion samples by newly developed three-dimensional impedance scanning electron microscopy**

##### Toshihiko Ogura^1*^, Tomoko Okada^1^

**^1^**Health and Medical Research Institute, National Institute of Advanced Industrial Science and Technology (AIST), Central 6, Higashi, Tsukuba, Ibaraki 305-8566, Japan

*Corresponding author: Toshihiko Ogura

Health and Medical Research Institute, National Institute of Advanced Industrial Science and Technology (AIST),

Higashi 1-1-1, Tsukuba, Ibaraki 305-8566, Japan

Tel.: +81-50-3521-2622,

E-mail: t-ogura@aist.go.jp

**This PDF file includes:**

Supplementary Figs 1 to 4 and Table 1


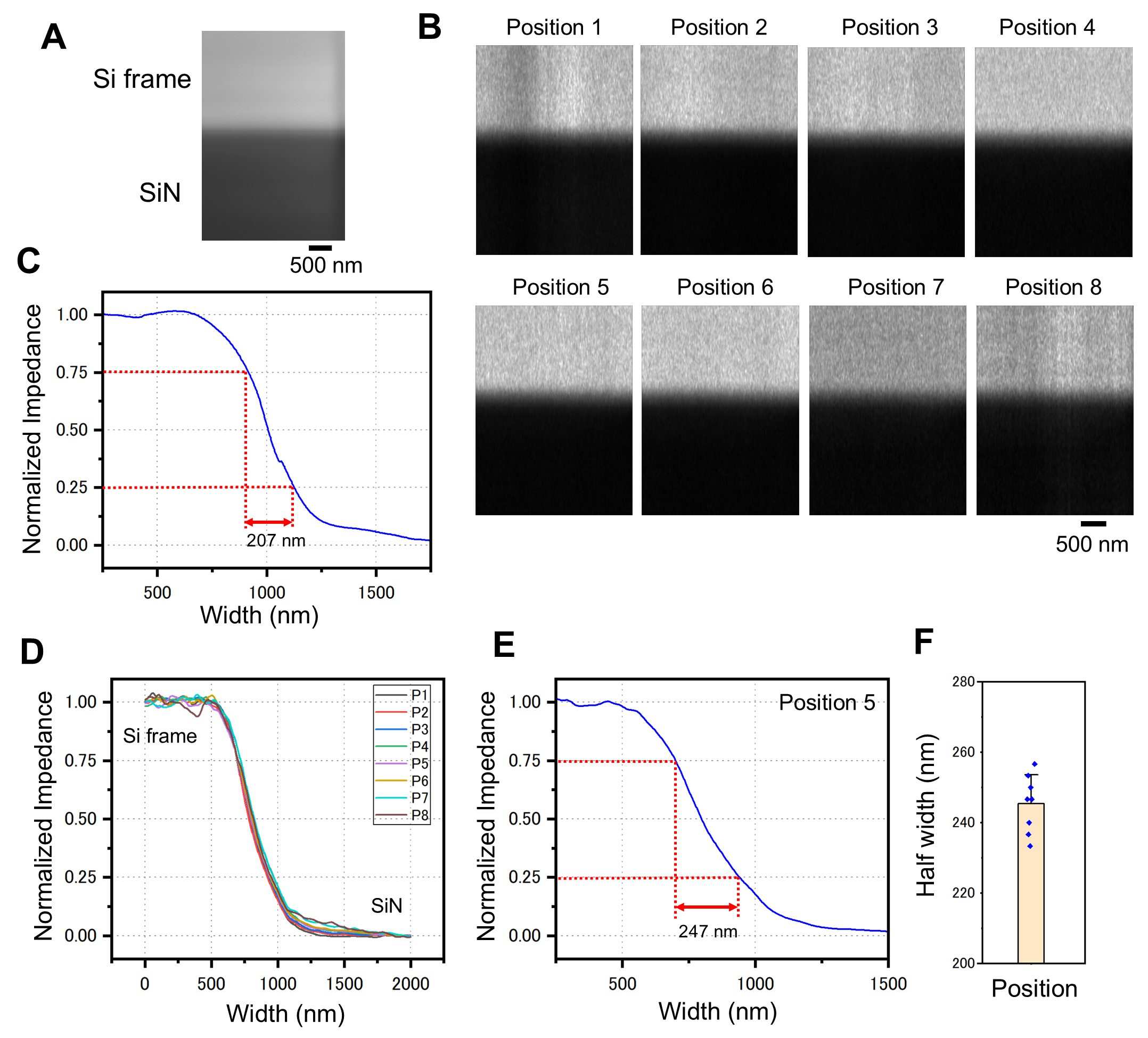


**Supplementary Fig. 1.** Measurement of spatial resolution of IP-SEM using SiN membrane and the edge of the Si frame**.** (A) Secondary electron image at the edge of the Si frame. (B) IP-SEM images of electrode positions 1 to 8. (C) Normalized line plot of the secondary electron image in (A). The half-width, which is an indication of spatial resolution of SEM, was 207 nm. (D) Normalized line plots of the IP-SEM image from the 8-electrode IP-SEM images. (E) Enlarged line plot of the edge of Position 5. The half-width, an indicator of spatial resolution, was 247 nm. (F) Graph of the half-width of eight IP-SEM images. The average value was 248 nm. Scale bars, 500 nm in (A) and (B).


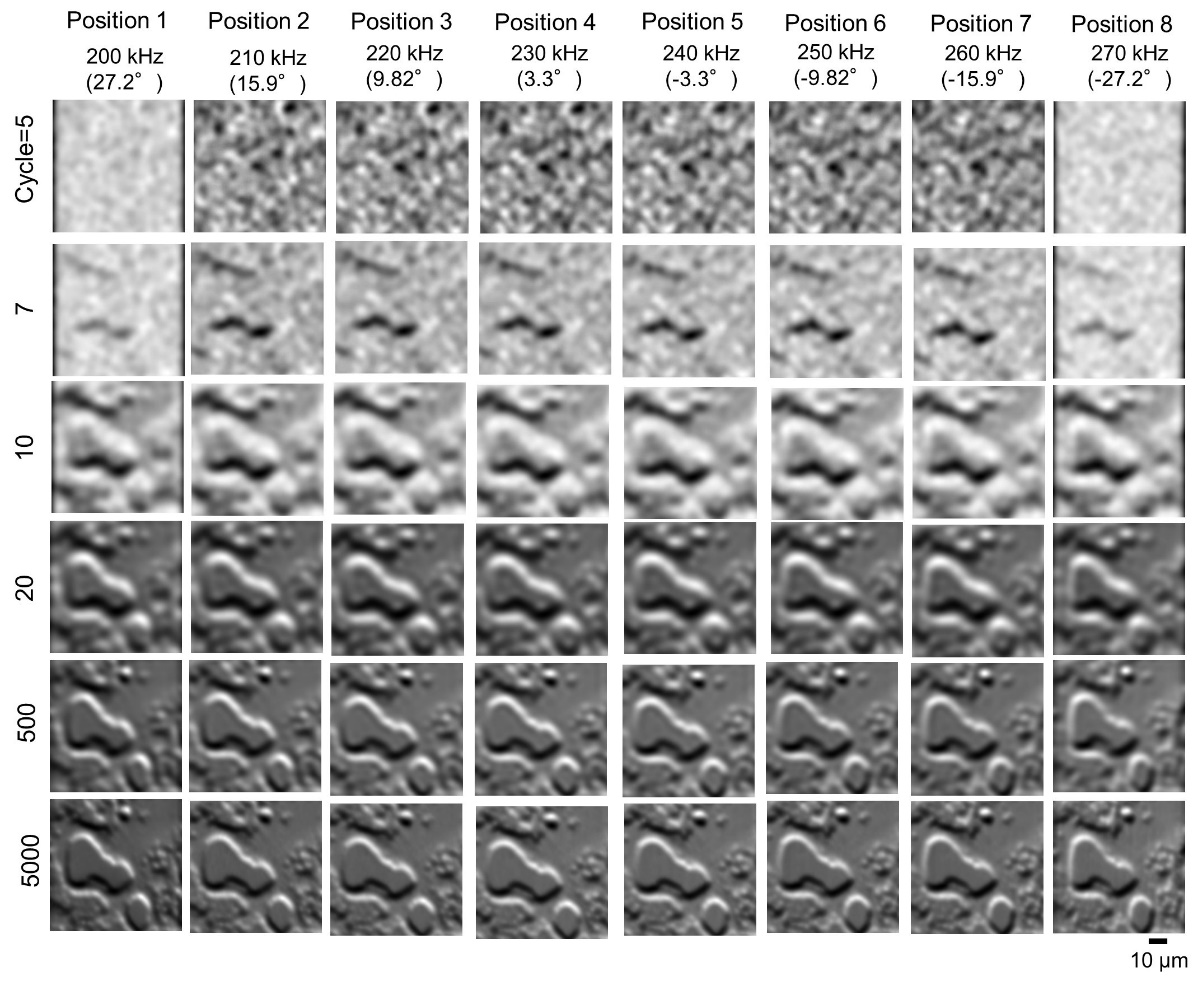


**Supplementary Fig. 2.** Each projection image during the calculation cycles using the SA 3D reconstruction method for sunscreen lotion images. Projection images for calculation cycles of 5, 7, 10, 20, 500, and 5000 are shown. This figure shows the intermediate projection images of all eight terminal positions in the calculation of Fig. 5.


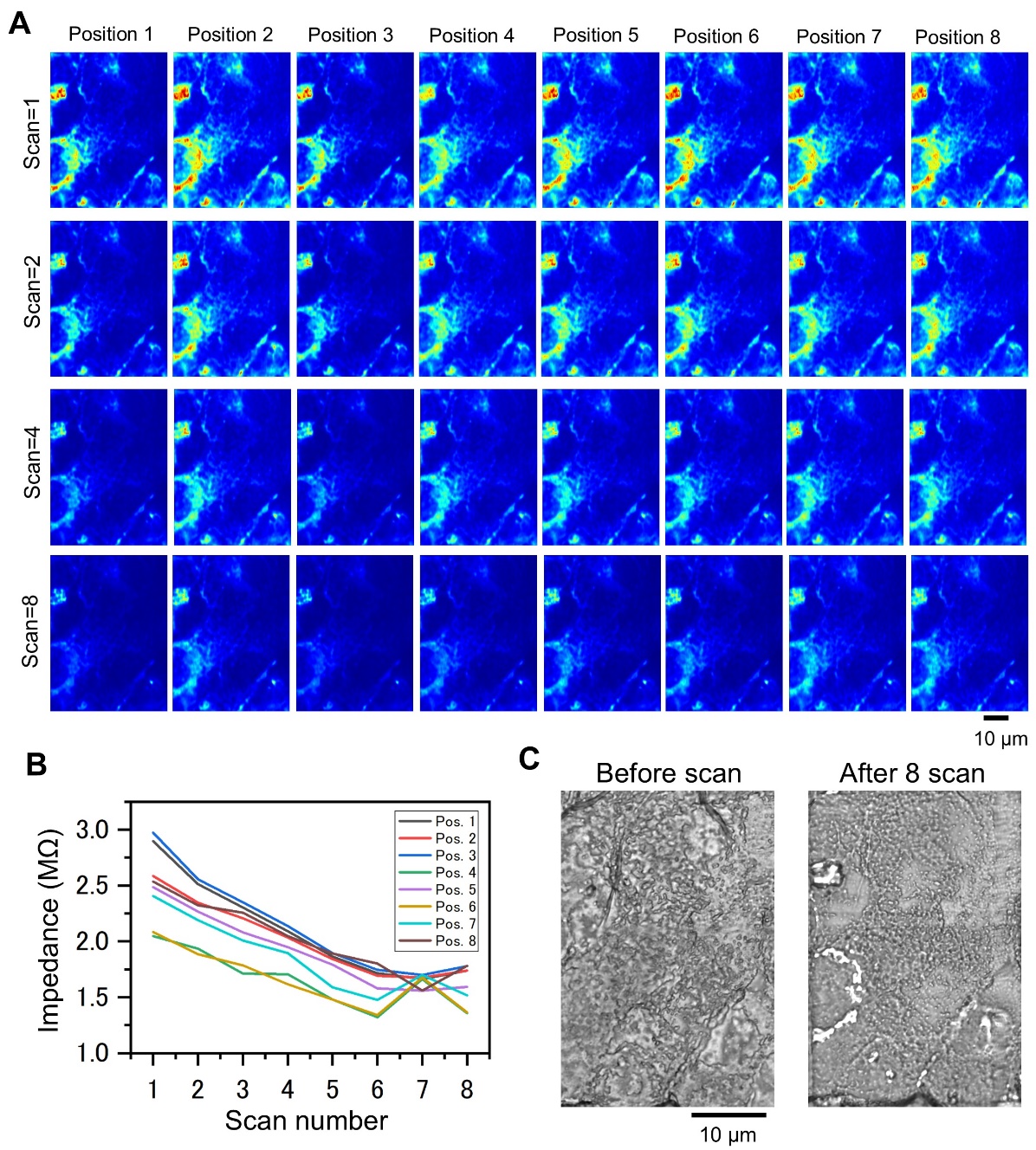


**Supplementary Fig. 3.** Changes in IP-SEM images when fixed and dried MNT-1 is scanned eight times. (A) Changes in impedance images using 8 electrodes after 1st to 8th EB scans. The cell structure is almost the same from the first scan to the second scan. However, from the 4th scan onwards, the contrast decreases considerably. (B) Changes in the average impedance value of each IP-SEM image depending on the number of scans. An almost linear decrease is observed until the 6th scan after which it becomes constant. (C) Optical microscope images of the observation area before and after eight scans. Scale bars, 10 μm in (A) and (C).


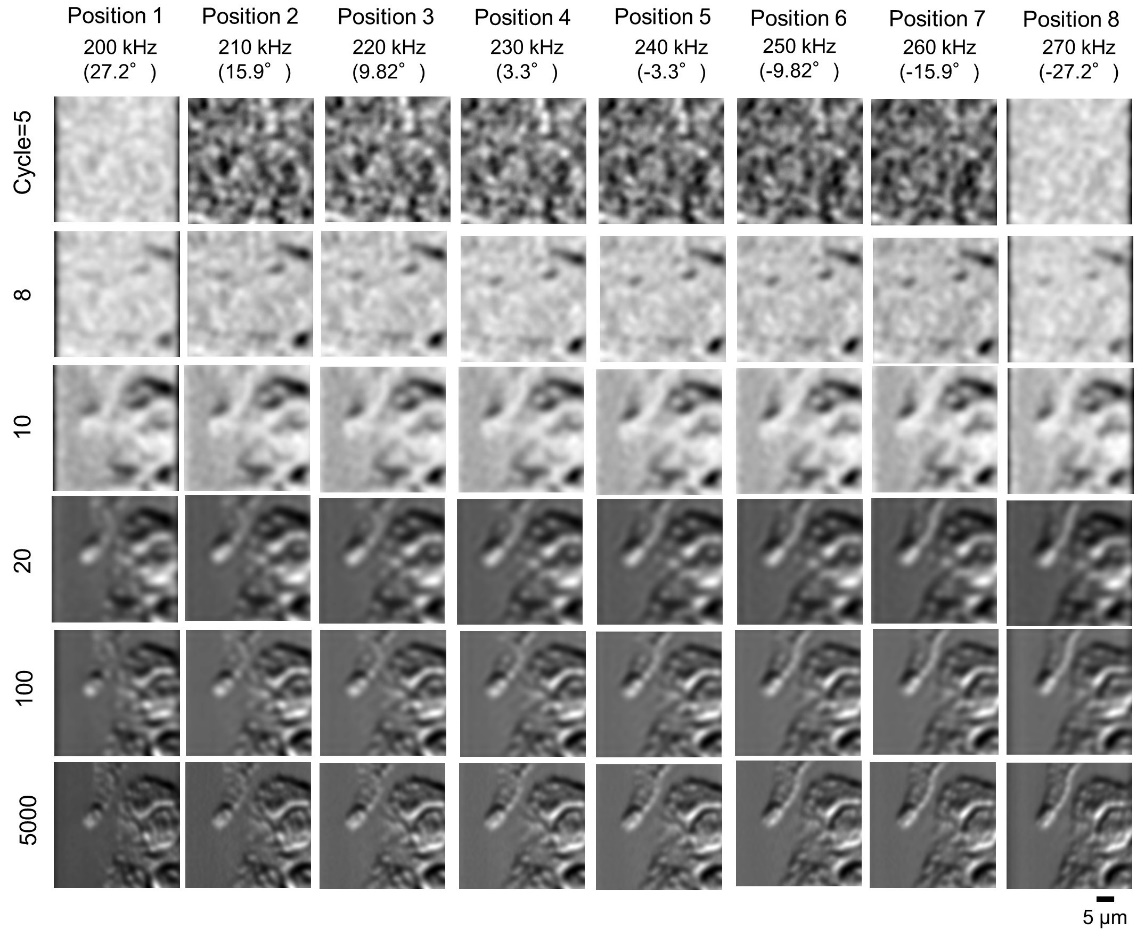


**Supplementary Fig. 4.** Each projection image during the calculation process using the SA 3D reconstruction method for MNT-1 cells. Projection images for calculation cycles of 5, 8, 10, 20, 100, and 5000 are shown. This figure shows the intermediate projection images of all eight terminal positions in the calculation of Fig. 8.


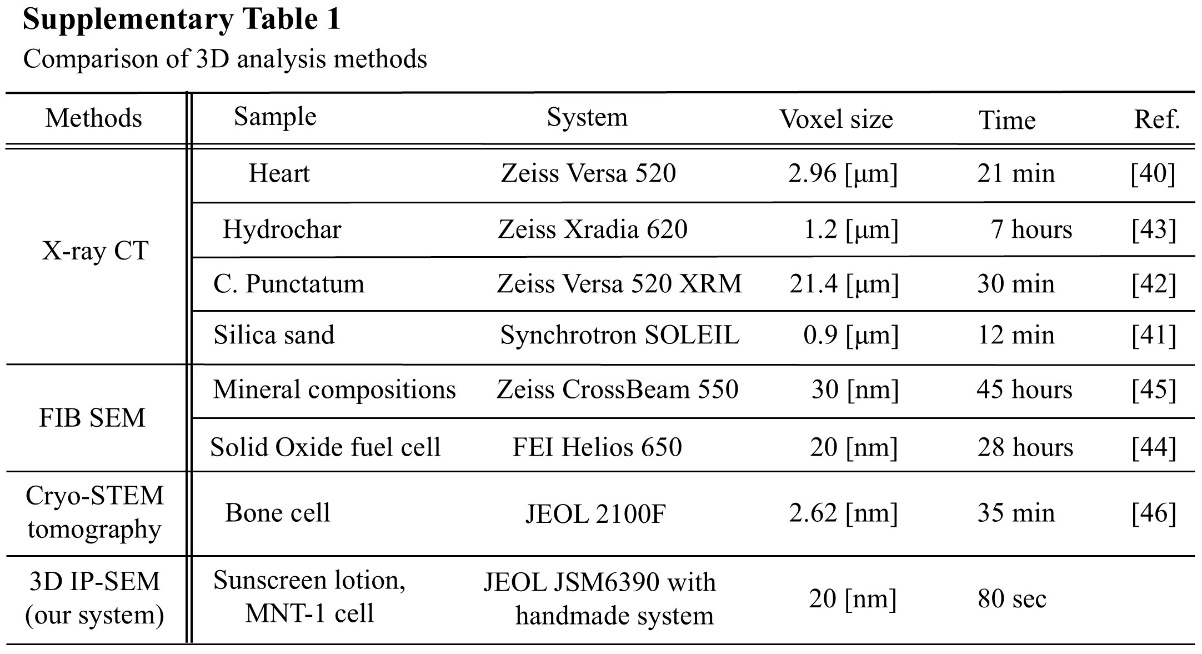

Supplement: Supplementary file 1 — Supplementary material [file mmc1.docx]
